# Association of coffee and caffeine consumption with risk and prognosis of endometrial cancer and its subgroups: a Mendelian randomization

**DOI:** 10.3389/fnut.2023.1291355

**Published:** 2023-11-14

**Authors:** Ziyu Chen, Chaosheng Liu, Jing Wu, Fandou Kong

**Affiliations:** ^1^Department of Gynecology and Obstetrics, The First Affiliated Hospital of Dalian Medical University, Dalian, Liaoning, China; ^2^Department of Cardiology, The First Affiliated Hospital of Dalian Medical University, Dalian, Liaoning, China

**Keywords:** endometrial cancer (EC), Mendelian randomization (MR), coffee consumption, caffeine consumption, endometrioid histology (EH)

## Abstract

**Background:**

Previous studies have not established potential causal associations between coffee and caffeine consumption in endometrial cancer (EC) and its subgroups. Therefore, we used a two-sample MR method to assess the causal association between coffee and caffeine consumption and EC risk. We also evaluated the association between these genetically predicted exposures and EC prognosis.

**Materials and methods:**

This study used 12 and two independent single-nucleotide polymorphisms (SNPs) associated with coffee and caffeine consumption as instrumental variables at a genome-wide significance level of *p* < 5 × 10^–8^. The EC Association Consortium (ECAC) performed a genome-wide association study (GWAS) analysis of 12,906 cases and 108,979 controls. FinnGen Consortium performed a GWAS analysis of 1,967 EC cases and 167,189 controls. The primary technique we employed was inverse-variance weighted, followed by the weighted median, MR-Egger regression, and MR robust adjusted profile score methods. We used the MR pleiotropy residual sum, Outlier test, and MR-Egger regression to assess Outlier and pleiotropic variants. We also conducted a sensitivity analysis through the leave-one-out method.

**Results:**

Genetically predicted coffee consumption was not associated with EC and its subgroups in the ECAC, and the association was consistent in the FinnGen consortium. After excluding eight SNPs with confounding factors, the study performed sensitivity analyses, delivering consistent results. We also observed that caffeine consumption was not correlated with EC risk. As confirmed by MR analysis, selected SNPs determined that most do not significantly impact the likelihood of developing EC.

**Conclusion:**

Our study indicated no convincing evidence supports coffee and caffeine consumption causing EC or impacting its prognosis. More studies are needed to validate the results.

## 1. Introduction

Endometrial cancer (EC) is one of the most common gynecologic malignancies. Its incidence was rising globally, with approximately 417,000 new cases in 2020 (1). If it continues its current trend, the number of women diagnosed with EC in the U.S. will reach 122,000 cases annually by 2030 (2).

It is recognized that Prolonged unopposed estrogen exposure is an established risk factor for EC. Metabolic factors such as obesity, insulin resistance, and dyslipidemia correlate with increased EC risk (3–6). Conversely, observational studies have shown that coffee and caffeine consumption negatively affect EC risk (7–9). Additionally, earlier research has linked higher coffee intake to lower levels of C-peptide and estrogen, two chemicals implicated in the development of endometrial cancer (10–12). However, the potential causal association of coffee and caffeine consumption with the risk of EC has yet to be established due to possible confounding factors and the lack of randomized controlled trials.

Mendelian randomization (MR) is a technique for assessing if an exposure factor has a causal effect on an outcome (13). MR strengthens causal inference by including genetic tools as exposure factors. It reduces reverse causation as alleles are randomly assigned during meiosis. The genetic tools are randomly assigned during conception and are usually not associated with confounding factors (14).

In this investigation, we evaluated the causal association of coffee and caffeine consumption with the risk of EC and its subgroups using a two-sample MR approach. We also assessed the correlation between the prognosis for EC and these genetically indicated exposures.

## 2. Materials and methods

### 2.1. Study design

**FIGURE 1:**
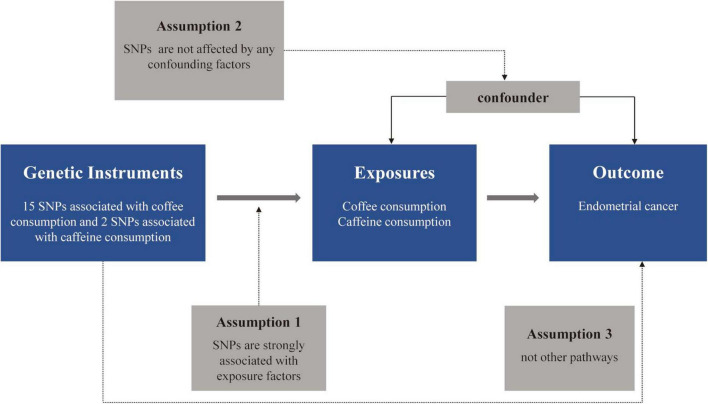
The flowchart of the Mendelian randomization (MR) study. SNP, single-nucleotide polymorphism.

Genetic variations serve as instrumental variables (IVs) in MR analyses to establish the causal link between exposure and outcome (15). MR analyses are focused on three essential hypotheses: (1) IVs are strongly associated with exposure factors; (2) IVs are not affected by any confounders; (3) IVs affect the outcome only through exposure factors, which are not related to the outcome (14). The flowchart of this MR study design is shown in Figure 1.

### 2.2. Genetic instrument selection

The 15 single-nucleotide polymorphisms (SNPs) correlated with coffee consumption, derived from a meta-analysis of four large-scale genome-wide association studies (GWASs), involved 375,833 individuals (UK Biobank and three US cohorts) of European descent (16) (Supplementary Table 1). The GWASs adjusted for sex, age, total energy, body mass index, and top 20 principal components. In the United Kingdom Biobank (discovery phase), a touch screen questionnaire was applied to collect coffee consumption from all participants at baseline: “How many cups of coffee do you drink each day (including decaffeinated coffee)?” In the United States cohorts (replication phase), a semi-quantitative food frequency questionnaire was used to collect the regular and decaffeinated coffee consumption (16). The effect sizes of SNP-coffee associations increased by 50% (equivalently from 1 cup to 1.5 cups). To fulfill the first MR hypothesis, we selected SNPs that were reliably genetically variables (*P* < 5 × 10^–8^) and independently (linkage disequilibrium; LD *r*^2^ < 0.001 and cluster window > 10,000 kb) (17, 18) associated with exposures. Meanwhile, we calculated the F statistic (*F* > 10 indicates sufficient instrumental strength) (14). To fulfill the second MR hypothesis, we assessed the pleiotropic relationship between SNPs and potential confounders by searching the PhenoScanner V2 website (19, 20). Finally, to fulfill the third MR hypothesis, we excluded SNPs with *P* < 0.05 to ensure that IVs were not associated with the outcome (14). In the preliminary analysis, 11 SNPs were used as IVs for coffee consumption. Due to potential genome-wide confounders, we excluded eight SNPs and the remaining three as IVs in the sensitivity analysis (Supplementary Table 2).

The two variants associated with caffeine consumption came from a meta-analysis of 6 GWAS and included 9,876 individuals of European ancestry (21) (Supplementary Table 1). A self-reported questionnaire was used to find out how much caffeine people in coffee, tea, and cola drank. Pooled data on SNP-caffeine correlations were obtained from GWAS of 4,460 females and scaled to increase the caffeine measure by 80 mg, approximately equal to a cup of coffee (22). IVs were consistent with a *P* < 5 × 10^–8^, independent, and strongly correlated with the F-statistic, and were used to perform MR analyses. Detailed information on SNPs related to coffee and caffeine consumption is shown in Table 1.

**TABLE 1:** Association of single nucleotide polymorphisms with coffee or caffeine consumption and endometrial cancer and its subtypes.

Exposure	SNP	EA	OA	EAF	Coffee or Caffeine Consumption			Endometrial cancer and its subtypes in ECAC									Endometrial cancer in FinnGen		
					Beta	SE	*P*	EC			EH			NEH			Beta	SE	*P*
								Beta	SE	*P*	Beta	SE	*P*	Beta	SE	*P*			
Coffee consumption	rs1057868	T	C	0.29	0.0197	0.0016	5.26E-33	–	–	–	0.0337	0.0198	0.09	0.0681	0.0477	0.15	0.0564	0.0333	0.09
	rs10865548	G	A	0.83	0.0154	0.0019	4.46E-15	0.0297	0.0203	0.14	0.0388	0.0239	0.10	−0.0296	0.0578	0.61	0.0386	0.0441	0.38
	rs1260326	C	T	0.61	0.0136	0.0015	2.62E-19	0.0288	0.0156	0.06	0.0293	0.0184	0.11	0.0121	0.0448	0.79	0.0294	0.0343	0.39
	rs1956218	G	A	0.56	0.0082	0.0015	3.62E-08	0.0141	0.0154	0.36	0.0273	0.0181	0.13	0.0413	0.0445	0.35	0.0225	0.0330	0.50
	rs2330783	G	T	0.99	0.0453	0.0063	1.57E-12	−0.0355	0.0654	0.59	−0.0401	0.0775	0.60	−0.1373	0.1796	0.44	−0.1951	0.1969	0.32
	rs2472297	T	C	0.27	0.0454	0.0017	5.19E-155	−0.0265	0.0181	0.14	−0.0146	0.0213	0.49	–	–	–	−0.0247	0.0379	0.51
	rs34060476	G	A	0.13	0.0189	0.0022	5.06E-18	–	–	–	0.0492	0.0285	0.08	−0.0583	0.0733	0.43	0.0897	0.0483	0.06
	rs4410790	C	T	0.63	0.0394	0.0015	5.59E-141	0.0203	0.0158	0.20	−0.0090	0.0186	0.63	−0.0242	0.0452	0.59	−0.0008	0.0345	0.98
	rs574367	T	G	0.21	0.0105	0.0018	8.06E-09	0.0030	0.0188	0.87	0.0069	0.0221	0.75	−0.0170	0.0545	0.75	0.0366	0.0422	0.39
	rs597045	A	T	0.69	0.0107	0.0015	6.62E-11	0.0009	0.0165	0.96	−0.0026	0.0199	0.90	0.0298	0.0503	0.55	–	–	–
	rs66723169	A	C	0.23	0.0147	0.0018	9.88E-17	0.0148	0.0181	0.41	0.0180	0.0213	0.40	0.0610	0.0515	0.24	0.0478	0.0428	0.26
Caffeine consumption	rs2470893	T	C	0.31	0.12	0.016	5.15E-14	−0.0241	0.0163	0.14	−0.0114	0.0192	0.55	−0.0910	0.0472	0.05	0.1356	0.1554	0.38
	rs4410790	T	C	0.62	0.15	0.017	2.36E-19	−0.0203	0.0158	0.20	0.0090	0.0186	0.63	0.0242	0.0452	0.59	−0.0547	0.1478	0.71

SNP, single-nucleotide polymorphism; EA, effect allele; OA, other allele; SE, standard error; EC, endometrial cancer; EH, endometrioid histology; NEH, non-endometrioid histology.

### 2.3. Data sources for endometrial cancer

Endometrial cancer-related data were obtained from the Endometrial Cancer Association Consortium (ECAC) and the FinnGen Consortium. ECAC performed a GWAS analysis of 12,906 cases and 108,979 controls (23). To avoid sample size overlap in the MR analysis, we removed the UK Biobank sample from the ECAC summary statistics, resulting in 12,270 EC cases and 46,126 controls (24). Furthermore, we analyzed the association of coffee and caffeine consumption with the risk of EC (8,758 patients with endometrioid histology and 1,230 cases with non-endometrioid histology) (25). We also performed Subgroup analyses in ECAC. GWAS analysis of 1,967 EC cases and 167,189 controls at the FinnGen Consortium (26). The ninth publication of the FinnGen Consortium database includes 377,277 individuals of Finnish ancestry, consisting of genes specific to the Finnish population, with high differential complementation accuracy and phenotypes from population-based registries. It includes cases from various disease domains, adjusted for age, sex, genetic principal components, and genotyping batches.

### 2.4. Data sources of BMI, smoking initiation, and alcohol consumption

Analyses were adjusted for differences in genetically predicted BMI, smoking initiation, and alcohol consumption using multivariable MR. The genetic variants linked to BMI and exposure variables were found through a GWAS meta-analysis in the Genetic Investigation of Anthropometric Traits^1^ consortium, which included 681,275 Europeans (27). GWAS data on 1,232,091 people showed summary-level information on how they started smoking (28). As was already said, GWAS data on 941,280 people showed that they drank alcohol, giving us summary-level statistics (28).

### 2.5. Statistical analysis

The inverse-variance weighted (IVW) was used as the primary statistical method. We used IVW with random effects to assess the relationships for genetically predicted coffee consumption. IVW fixed-effects models (analyses with several SNPs less than three) were used to estimate the interactions between genetic prediction and caffeine consumption. We also performed the weighted median (WM) and MR-Egger regression methods (29). The IVW method is applied to assume that all SNPs are valid and independent, and its meta-aggregation of multiple side effects in MR analyses of numerous SNPs (30). WM is the median obtained after weighting individual SNPs (31). The WM approach provides robust estimates, with at least 50% of the information coming from valid instrumental variables (32). The MR-Egger method allows for the inclusion of instrumental variables with a multivariate effect if the intercept *P*-value < 0.05 indicates the presence of horizontal multivariate validity (33). We applied the MR-pleiotropy residual sum and outlier (MR-PRESSO) methods to detect the presence of horizontal pleiotropy, and Cochran’s Q statistic was used to assess heterogeneity between SNPs in each analysis (34). We also conducted a sensitivity analysis through the leave-one-out method.

Moreover, we assessed the pathogenic impact of the selected SNPs on EC prognosis by MEndelian Randomization (SUMMER^2^), by the framework of MR analysis based on the Multi-Organomics Database of SUrvival-Related Cancers (35). All analyses were performed using the “TwoSampleMR” and “MR-PROESSO” packages in R software (version 4.3.1), and all statistical tests were two-sided.

## 3. Results

### 3.1. Causal relationship between coffee and caffeine consumption with risk of EC and its subtypes

The F-statistics for coffee (the 50% increase) and caffeine (the per 80-mg increase) in this study were 159 and 67, indicating that the IVs had sufficient instrumental strength. Furthermore, MR-PRESSO did not detect abnormal SNPs in preliminary and sensitivity analyses. We did not detect heterogeneity by IVW and MR-Egger regression.

**FIGURE 2:**
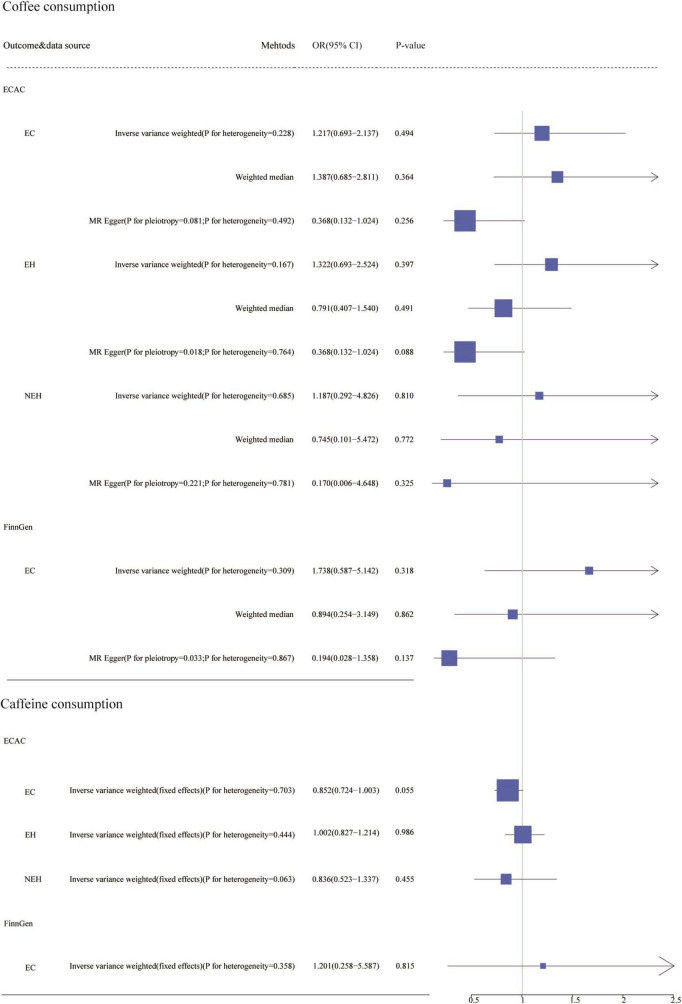
The association of genetically predicted coffee and caffeine consumption with endometrial cancer (EC) and its Subgroups. ECAC, the Endometrial Cancer Association Consortium; EH, Endometrioid histology; NEH, non-endometrioid histology; FinnGen, FinnGen Consortium; OR, odds ratio; CI, confidence interval.

**FIGURE 3:**
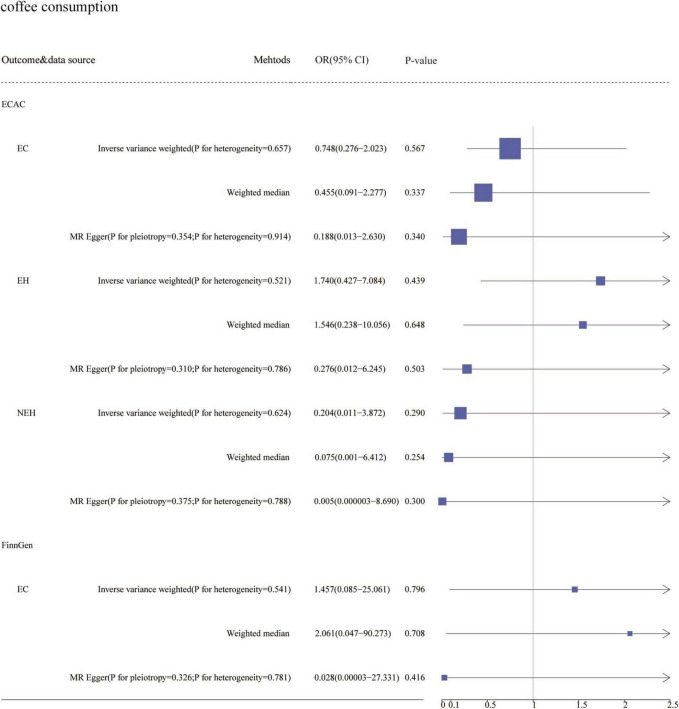
Results of sensitivity analyses association of genetically predicted coffee consumption with EC. ECAC, the Endometrial Cancer Association Consortium; EH, Endometrioid histology; NEH, non-endometrioid histology; FinnGen, FinnGen Consortium; OR, odds ratio; CI, confidence interval.

In preliminary analyses, we found pleiotropy (*p* < 0.05) in the causal relationship between two data sets and coffee consumption by MR-Egger: endometrioid endometrial carcinoma (EEC) in the ECAC consortium and EC in the FinnGen consortium (Figure 2). To investigate the causality between coffee consumption and the risk of both EC and its subtypes, we processed the ECAC data by random-effects IVW. We found that coffee consumption was not associated with EC (OR = 1.217, 95% CI: 0.693–2.137). We also conducted a subgroup analysis of the ECAC data, which suggested that coffee consumption was not linked to non-endometrioid endometrial carcinoma (NEC) (OR = 1.187, 95% CI: 0.292–4.826). Furthermore, in the FinnGen consortium, coffee consumption did not affect EC (OR = 1.738, 95% CI: 0.587–5.142) (Figure 2). In sensitivity analyses, we did not find directed pleiotropy (*p* > 0.05). Additionally, we performed random-effects IVW analyses on the ECAC data and FinnGen consortium data, resulting in no significant differences from the preliminary analyses (Figure 3). Simultaneously, we adjusted pertinent variables such as body mass index, smoking initiation, and alcohol consumption and discovered that the outcomes did not deviate significantly from those of the primary analysis (Supplementary Table 3).

To investigate the causal relationship between caffeine consumption and established outcomes, we analyzed the ECAC data by fixed-effects IVW, which showed that caffeine consumption was unrelated to the risk of EC (OR = 0.852, 95% CI: 0.852–1.003). We also performed subgroup analyses, which showed that caffeine consumption was not a factor in the pathogenesis of EEC (OR = 1.002, 95% CI: 0.827–1.214) and NEC (OR = 0.836, 95% CI: 0.523–1.337). In the FinnGen Consortium data analysis, we also observed that caffeine consumption was not correlated with EC risk (OR = 1.201, 95% CI: 0.258–5.587) (Figure 2).

The study also performed a leave-one-out analysis, which excluded the effect of individual SNPs on the overall causal estimate by removing each SNP stepwise and repeating the MR analysis. The leave-one-out analysis showed relatively stable results after removing each SNP (Supplementary Figures 1–4).

### 3.2. Effect of coffee and caffeine consumption on EC prognosis

**FIGURE 4:**
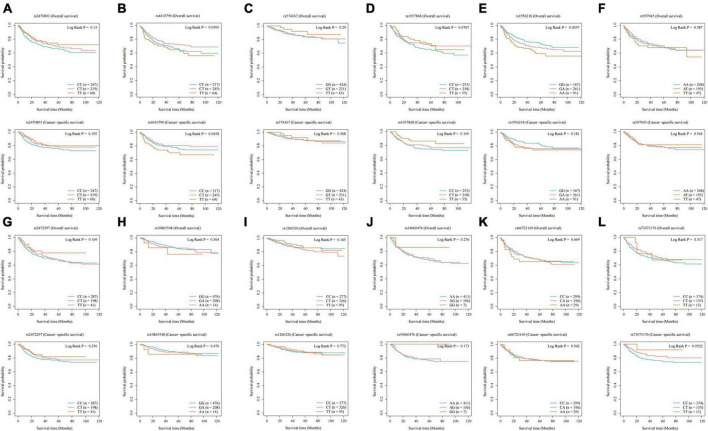
Kaplan–Meier plots of the effect of coffee and caffeine consumption on overall survival and cancer-specific survival in EC. **(A)** rs2470893; **(B)** rs4410790; **(C)** rs574367; **(D)** rs1057868; **(E)** rs1956218; **(F)** rs597045; **(G)** rs2472297; **(H)** rs10865548; **(I)** rs1260326; **(J)** rs34060476; **(K)** rs66723169; **(L)** rs73073176.

Our analysis of selected SNPs has determined that most do not significantly impact the likelihood of developing EC, as confirmed by MR analysis. Shorter overall survival (OS) in EC was positively associated with the SNPs rs1956218 (HR: 1.275, *P* = 0.036) linked to coffee consumption, while the SNPs rs4410790 (HR: 0.702, *P* = 0.022) had the opposite effect. Additionally, coffee- and caffeine-consumption-associated SNPs rs4410790 (HR: 0.632, *P* = 0.012) and caffeine-consumption-associated SNPs rs73073176 (HR: 0.652, *P* = 0.048) were also identified to be associated with shorter cancer-specific survival (CSS) (Table 2; Figure 4).

**TABLE 2:** Effect of coffee and caffeine consumption on overall survival and cancer-specific survival in all endometrial cancers.

SNP	Exposure	HR_OS	SE_OS	P_value_OS	HR_CSS	SE_CSS	P_value_CSS
rs4410790	Caffeine consumption coffee consumption	0.702	0.154	0.022	0.632	0.182	0.012
rs2470893	Caffeine consumption	1.005	0.145	0.974	1.129	0.166	0.464
rs574367	Coffee consumption	0.908	0.168	0.565	0.991	0.189	0.960
rs10865548	Coffee consumption	1.108	0.193	0.596	1.099	0.222	0.672
rs1260326	Coffee consumption	1.112	0.147	0.471	1.067	0.172	0.708
rs73073176	Coffee consumption	0.878	0.159	0.412	0.652	0.216	0.048
rs34060476	Coffee consumption	0.869	0.181	0.438	0.800	0.228	0.329
rs1057868	Coffee consumption	0.827	0.120	0.114	0.797	0.150	0.130
rs597045	Coffee consumption	1.026	0.122	0.836	1.018	0.153	0.906
rs1956218	Coffee consumption	1.275	0.116	0.036	1.254	0.144	0.116
rs2472297	Coffee consumption	0.869	0.126	0.266	0.834	0.157	0.246
rs66723169	Coffee consumption	1.037	0.130	0.779	0.966	0.162	0.830

SNP, single-nucleotide polymorphism; HR, hazard ratio; OS, overall survival; CSS, cancer-specific survival.

## 4. Discussion

Our study did not find genetically predicted associations between coffee and caffeine consumption regarding the risk of EC and its subgroups. No outlier SNPs were detected, although preliminary analyses detected pleiotropy in individual groups. Leave-one-out analyses also showed relatively stable results. After excluding SNPs with confounding factors, the study performed sensitivity analyses that did not detect pleiotropy or heterogeneity, delivering consistent results. Furthermore, we found that most SNPs were not associated with EC prognosis by MR analysis.

Previous research has been controversial regarding the association between coffee or caffeine consumption and the risk of EC. In recent times, a cross-sectional study (36) demonstrated that caffeine was not associated with the risk of EC [OR, 95% CI; 0.999 (0.996, 1.001), *P* = 0.297]. Moreover, a large prospective study (37) investigating the relationship between coffee and EC risk found that coffee intake was not significantly associated with EC risk. A published meta-analysis that resulted in this study also found a weak association between coffee consumption and EC risk. However, a meta-analysis including six cohort studies and 13 case-control studies supported coffee consumption’s potentially beneficial health effects on EC, especially in women with higher BMI (38). Meanwhile, in a meta-analysis of observational studies by Je et al. (39), an increase in coffee consumption of one cup/day was negatively associated with the risk ratio of EC and similar findings were reported by Yang et al. (37), Lafranconi et al. (40) and Lukic et al. (41). Despite this, most of the results supported that coffee and caffeine consumption were associated with a reduced risk of EC. However, these findings didn’t indicate that coffee and caffeine consumption was responsible for the reduced risk of EC. Due to methodological constraints and residual confounders, observational studies might only partially account for some factors influencing a result (such as the effects of a healthy lifestyle and diet).

Recently, there have been large-scale Mendelian randomization studies on coffee consumption and overall cancers, including EC, reported no causal relationship (42, 43). Nevertheless, our investigation not only examined the potential causal association of coffee and caffeine consumption with the risk of EC, but also assessed its impact on the relationship between EC progression. Confounders did not influence two-sample MR analyses, and we reduced reverse causality by using genetic variation as an instrumental variable. In this study, we assessed the association of selected SNPs with OS and CSS and produced Kaplan-Meier plots to illustrate. In terms of MR analysis, we applied two independent populations (ECAC and FinnGen consortium) separately, and the broadly consistent results ensured stability.

The study also has several disadvantages. Individual groups had horizontal pleiotropy in preliminary analyses, yet after excluding SNPs with potential confounders, we performed sensitivity analyses with generally consistent results. Second, most studies on coffee and caffeine used self-report methodologies, which were prone to bias. In addition, in this study, we did not stratify the menopausal status of EC patients, which might have led to the effect of coffee and caffeine intake on the risk of OC being influenced by menopausal status. Our studies were based on European populations and may need to be more generalizable to others.

## 5. Conclusion

Our MR investigation found no persuasive evidence to indicate a causal relationship between coffee and caffeine consumption and the risk of EC, and it was found to be largely irrelevant to the prognosis of EC. In the future, more clinical and basic studies are still needed to validate our results.

## Data availability statement

The original contributions presented in this study are included in this article/Supplementary material, further inquiries can be directed to the corresponding author.

## Author contributions

ZC: Conceptualization, Data curation, Investigation, Methodology, Software, Validation, Writing—original draft. CL: Investigation, Methodology, Software, Supervision, Validation, Writing—original draft. JW: Conceptualization, Investigation, Validation, Visualization, Writing—original draft. FK: Formal analysis, Methodology, Project administration, Resources, Writing—review and editing.

## Footnotes

1 https://portals.broadinstitute.org/collaboration/giant/index.php/GIANT_consortium_data_files (accessed April 28, 2021).

2
http://njmu-edu.cn:3838/SUMMER/

## Supplementary material

Supplementary Figure 1 Leave-one-out method of studies investigating the association between EC and coffee consumption in ECAC.

Supplementary Figure 2 Leave-one-out method of studies investigating the association between EH and coffee consumption in ECAC.

Supplementary Figure 3 Leave-one-out method of studies investigating the association between NEH and coffee consumption in ECAC.

Supplementary Figure 4 Leave-one-out method of studies investigating the association between EC and coffee consumption in FinnGen Consortium.
