# Supplementary material for: Association of coffee and caffeine consumption with risk and prognosis of endometrial cancer and its subgroups: a Mendelian randomization
Source: Front Nutr. 2023 Nov 14;10:1291355. doi: 10.3389/fnut.2023.1291355 (PMC10682782; doi:10.3389/fnut.2023.1291355)
Supplement: Supplementary file 1 [file Table_1.docx]

Supplementary Table 1. Information of included studies and consortia.

|  | Study or consortium | participants |
| --- | --- | --- |
| Coffee consumption | Zhong VW | 375,833 European-ancestry individuals |
| Caffeine consumption | Cornelis MC | 9,876 European-ancestry individuals |
| Endometrial cancer | The Endometrial Cancer Association Consortium (ECAC) | 12,906 European-ancestry cases and 108,979 European-ancestry controls |
| Endometrial cancer (endometrioid histology) | The Endometrial Cancer Association Consortium (ECAC) | 8,758 European-ancestry cases and 46,126 European-ancestry controls |
| Endometrial cancer (Non-endometrioid histology) | The Endometrial Cancer Association Consortium (ECAC) | 1,230 European-ancestry cases and 35,447 European-ancestry controls |
| Endometrial cancer | The Finngen Consortium | - |
