# Supplementary material for: Association of coffee and caffeine consumption with risk and prognosis of endometrial cancer and its subgroups: a Mendelian randomization
Source: Front Nutr. 2023 Nov 14;10:1291355. doi: 10.3389/fnut.2023.1291355 (PMC10682782; doi:10.3389/fnut.2023.1291355)
Supplement: Supplementary file 2 [file Table_2.docx]

Supplementary Table 2. Potential secondary phenotypes of the genetic variants used for coffee consumption.

| SNP | Trait |
| --- | --- |
| rs2472297 | Creatinine in urine |
|  | Leg fat percentage left |
|  | Leg fat percentage right |
|  | Impedance of arm right |
|  | Potassium in urine |
|  | Platelet distribution width |
| rs66723169 | Body mass index |
|  | Alcohol intake frequency |
|  | Arm fat mass left |
|  | Arm fat mass right |
|  | Comparative body size at age 10 |
|  | Comparative height size at age 10 |
|  | Forced expiratory volume in 1-second, predicted |
|  | Height |
|  | Hip circumference |
|  | Impedance of arm left |
|  | Impedance of arm right |
|  | Impedance of leg left |
|  | Impedance of leg right |
|  | Impedance of whole body |
|  | Leg fat mass left |
|  | Leg fat mass right |
|  | Leg fat percentage left |
|  | Leg fat percentage right |
|  | Leg fat-free mass left |
|  | Leg fat-free mass right |
|  | Leg predicted mass left |
|  | Leg predicted mass right |
|  | Waist circumference |
|  | Whole body water mass |
|  | Worrier or anxious feelings |
|  | Coronary artery disease |
| rs574367 | Body mass index |
|  | Childhood body mass index |
|  | Body mass index in female non-smokers |
|  | Body mass index in non-smokers |
|  | Body mass index in smokers |
|  | Age at menarche |
| rs1260326 | Granulocyte count |
|  | Granulocyte percentage of myeloid white cells |
|  | High light scatter percentage of red cells |
|  | High light scatter reticulocyte count |
|  | log eGFR creatinine |
|  | Type II diabetes |
|  | Height |
|  | Total cholesterol |
|  | Triglycerides |
|  | 2-hour glucose |
|  | Albumin |
|  | C reactive protein |
|  | FVII activity |
|  | FVII in plasma |
|  | Fasting blood glucose |
|  | Fasting insulin |
|  | Gamma glutamyl transferase |
|  | HDL cholesterol mean size lipoprotein fraction concentration |
|  | Hypertriglyceridemia |
|  | IFT172 expression in Lymphocytes lymphoblastoid cell lines tissue |
|  | IFT172 gene expression in adipose tissue |
|  | Serum creatinine estimated glomerular filtration rate eGFR |
|  | Serum urate |
|  | Triglycerides |
|  | Uric acid |
|  | Alcohol consumption |
|  | Cardiovascular disease risk factors |
| rs10865548 | Body mass index females |
|  | Body mass index males |
|  | Body mass index |
|  | Nonsyndromic striae distensae stretch marks |
|  | Age at menarche |
|  | Arm fat mass left |
|  | Arm fat mass right |
|  | Arm fat percentage left |
|  | Arm fat percentage right |
|  | Arm fat-free mass left |
|  | Arm fat-free mass right |
|  | Weight |
|  | Whole body fat mass |
|  | Whole body fat-free mass |
|  | Whole body water mass |
| rs4410790 | Habitual caffeine consumption |
|  | Habitual caffeine consumption caffeinated coffee intake |
|  | Habitual caffeine consumption female |
|  | Habitual caffeine consumption never smokers |
|  | Creatinine in urine |
|  | Potassium in urine |
|  | Sodium in urine |
| rs34060476 | Granulocyte percentage of myeloid white cells |
|  | High light scatter percentage of red cells |
|  | High light scatter reticulocyte count |
|  | Monocyte percentage of white cells |
|  | Reticulocyte count |
|  | Reticulocyte fraction of red cells |
|  | Triglycerides |
|  | Arm fat-free mass left |
|  | Arm fat-free mass right |
|  | Arm predicted mass left |
|  | Arm predicted mass right |
|  | Basal metabolic rate |
|  | Hip circumference |
|  | Impedance of arm left |
|  | Impedance of arm right |
|  | Impedance of leg right |
|  | Impedance of whole body |
|  | Leg fat-free mass left |
|  | Leg fat-free mass right |
|  | Leg predicted mass left |
|  | Leg predicted mass right |
|  | Self-reported gout |
|  | Sitting height |
|  | Sodium in urine |
| rs1057868 | Mean platelet volume |
|  | Creatinine in urine |
