# Supplementary material for: Association of coffee and caffeine consumption with risk and prognosis of endometrial cancer and its subgroups: a Mendelian randomization
Source: Front Nutr. 2023 Nov 14;10:1291355. doi: 10.3389/fnut.2023.1291355 (PMC10682782; doi:10.3389/fnut.2023.1291355)
Supplement: Supplementary file 3 [file Table_3.docx]

| Consortiums | Important factors | Methods | OR(95% CI) | P-value |
| --- | --- | --- | --- | --- |
| ECAC | Body mass index | Inverse variance weighted(P for heterogeneity<0.001) | 0.969(-0.185,0.123) | 0.693 |
|  |  | MR Egger(P for pleiotropy=0.047;P for heterogeneity<0.001) | 1.398(-0.058,0.729) | 0.509 |
|  | Smoking initiation | Inverse variance weighted(P for heterogeneity=0.0406) | 1.089(-0.051,0.221) | 0.219 |
|  |  | MR Egger(P for pleiotropy=0.034;P for heterogeneity=0.060) | 0.643(-0.949,0.065) | 0.137 |
|  | Alcohol consumption | Inverse variance weighted(P for heterogeneity<0.001) | 0.704(-0.815,0.112) | 0.494 |
|  |  | MR Egger(P for pleiotropy<0.001;P for heterogeneity=0.009) | 8.174(0.729,3.472) | 0.003 |
| FinnGen | Body mass index | Inverse variance weighted(P for heterogeneity=0.556) | 1.139(-0.126,0.386) | 0.320 |
|  |  | MR Egger(P for pleiotropy=0.458;P for heterogeneity=0.549) | 0.909(-0.743,0.552) | 0.773 |
|  | Smoking initiation | Inverse variance weighted(P for heterogeneity=0.409) | 0.985(-0.291,0.260) | 0.913 |
|  |  | MR Egger(P for pleiotropy=0.471;P for heterogeneity=0.399) | 0.685(-1.406,0.647) | 0.469 |
|  | Alcohol consumption | Inverse variance weighted(P for heterogeneity=0.796) | 0.778(-1.000,0.498) | 0.511 |
|  |  | MR Egger(P for pleiotropy=0.098;P for heterogeneity=0.841) | 4.276(-0.702,3.608) | 0.186 |

Supplementary Table 3. Association of single nucleotide polymorphisms with coffee consumption and endometrial cancer adjusted by body mass index, smoking, and alcohol consumption.

ECAC, the Endometrial Cancer Association Consortium; FinnGen, FinnGen Consortium; OR, odds ratio; CI, confidence interval.
