# Supplementary figures and images for: Association of coffee and caffeine consumption with risk and prognosis of endometrial cancer and its subgroups: a Mendelian randomization
Source: Front Nutr. 2023 Nov 14;10:1291355. doi: 10.3389/fnut.2023.1291355 (PMC10682782; doi:10.3389/fnut.2023.1291355)

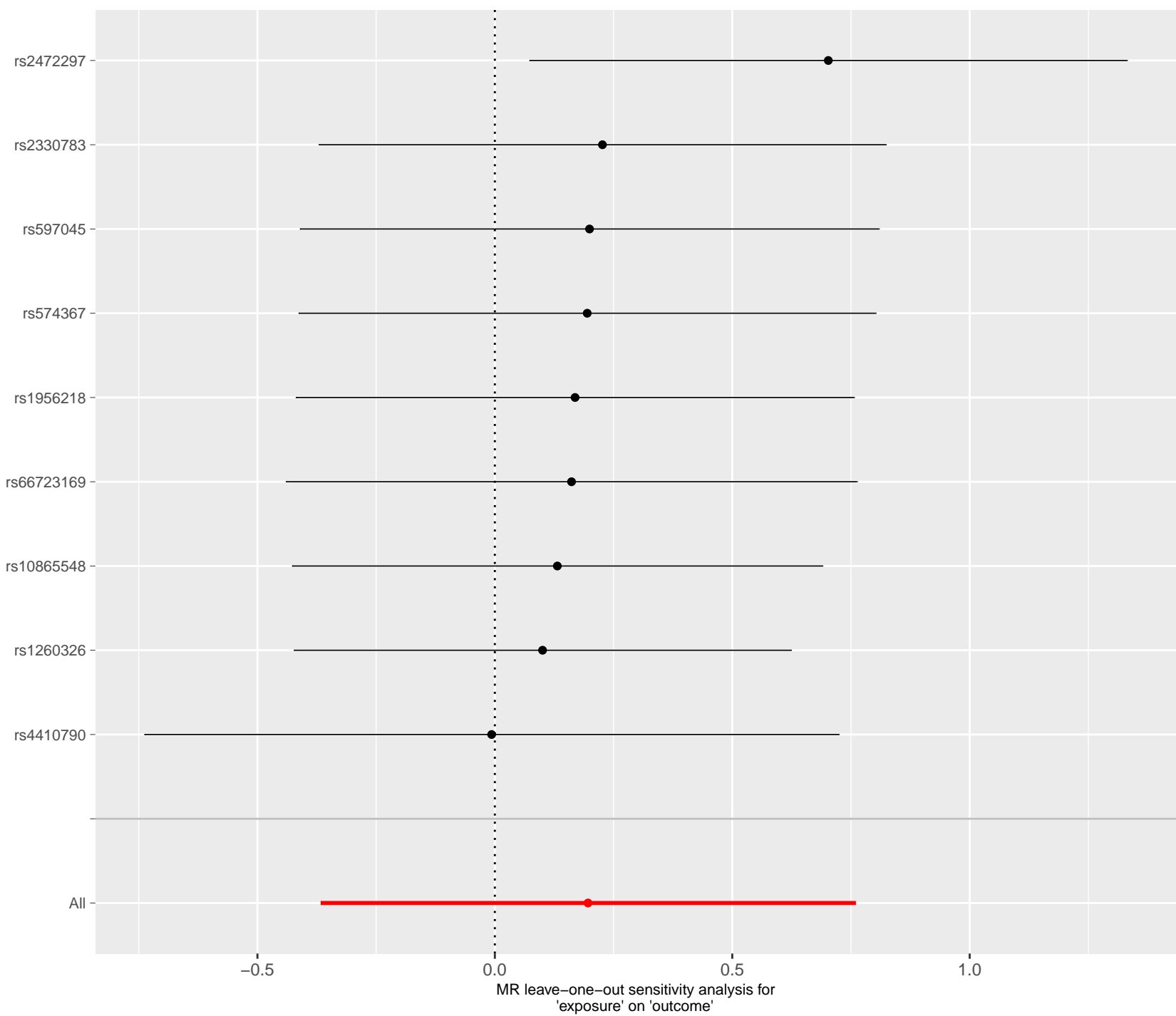

Supplement: Supplementary Figure 1 — Leave-one-out method of studies investigating the association between EC and coffee consumption in ECAC. [file Data_Sheet_1.PDF]

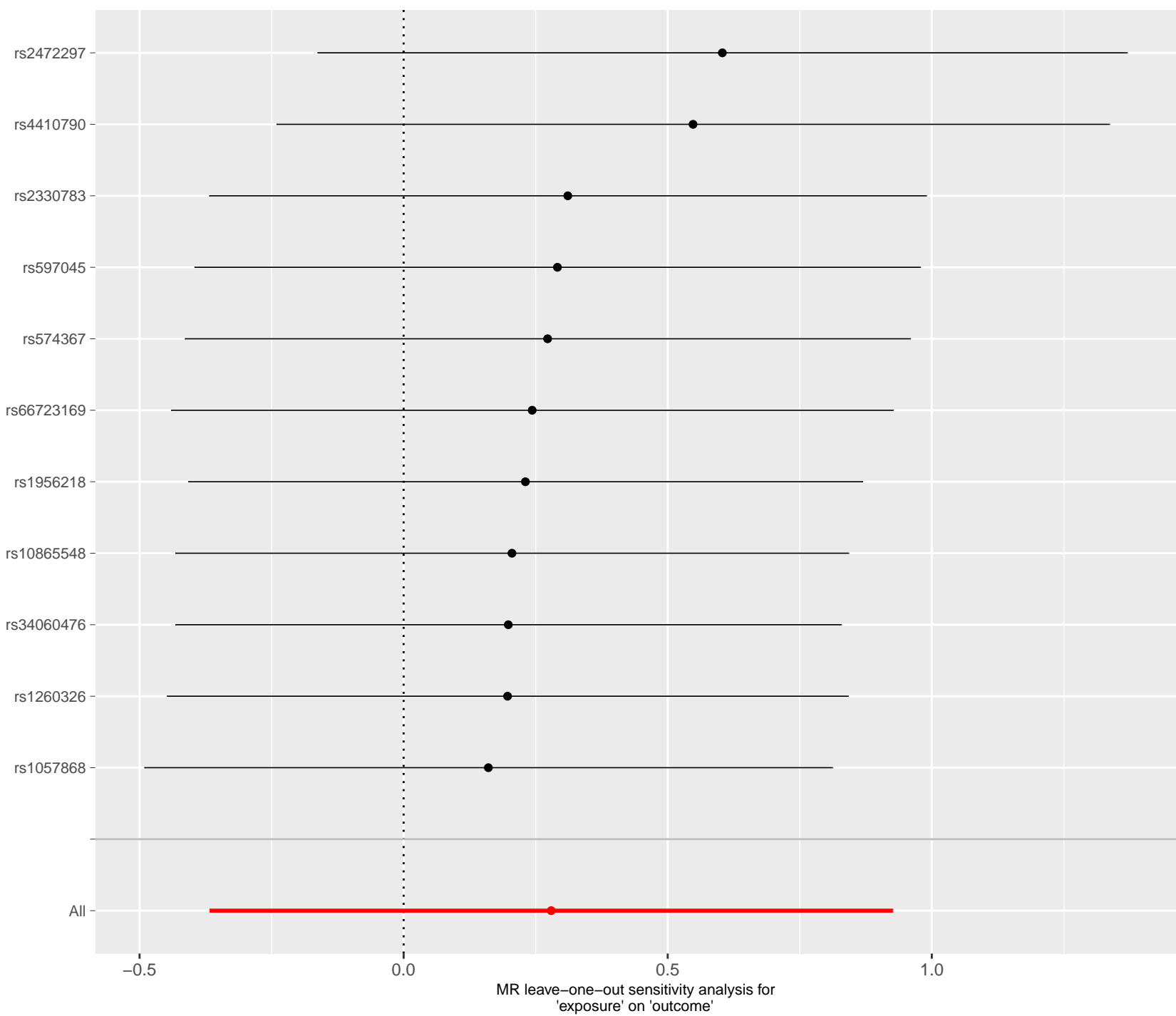

Supplement: Supplementary Figure 2 — Leave-one-out method of studies investigating the association between EH and coffee consumption in ECAC. [file Data_Sheet_2.PDF]

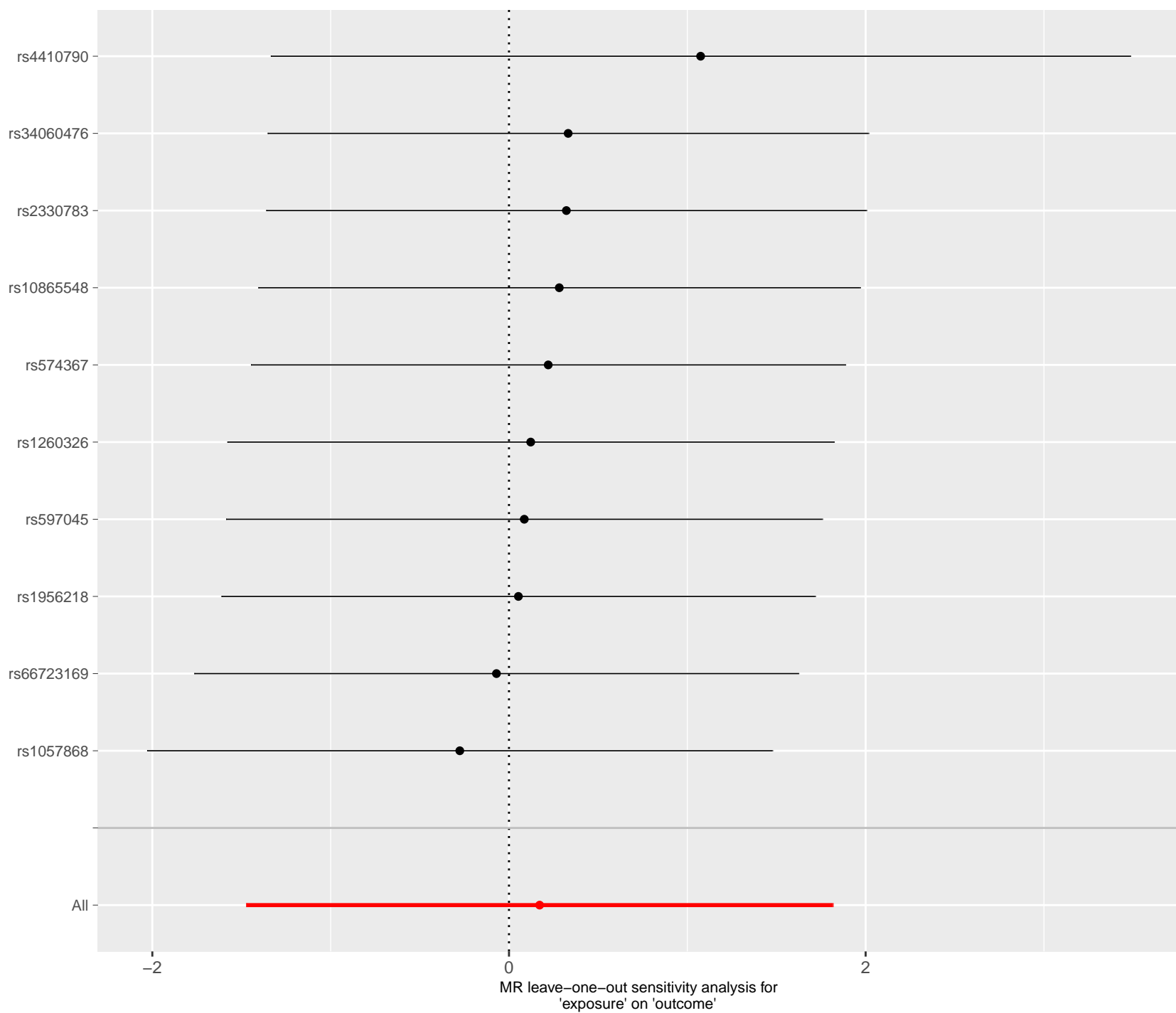

Supplement: Supplementary Figure 3 — Leave-one-out method of studies investigating the association between NEH and coffee consumption in ECAC. [file Data_Sheet_3.PDF]

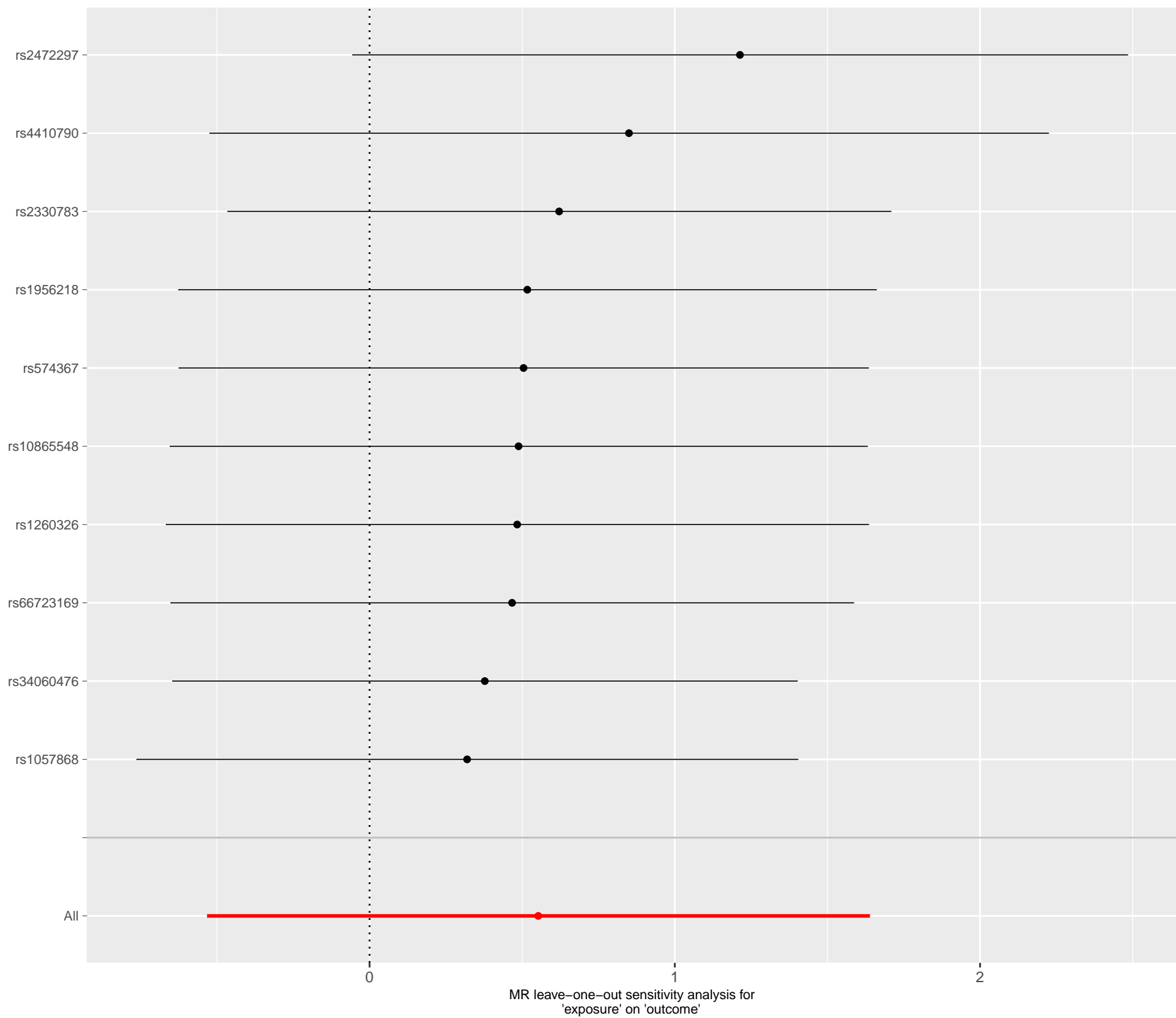

Supplement: Supplementary Figure 4 — Leave-one-out method of studies investigating the association between EC and coffee consumption in FinnGen Consortium. [file Data_Sheet_4.PDF]
